# Environmental Factors Influencing Occurrence of Vibrio parahaemolyticus and Vibrio vulnificus

**DOI:** 10.1128/aem.00307-23

**Published:** 2023-05-24

**Authors:** Kyle D. Brumfield, Arlene J. Chen, Mayank Gangwar, Moiz Usmani, Nur A. Hasan, Antarpreet S. Jutla, Anwar Huq, Rita R. Colwell

**Affiliations:** a Maryland Pathogen Research Institute, University of Maryland, College Park, Maryland, USA; b University of Maryland Institute for Advanced Computer Studies, University of Maryland, College Park, Maryland, USA; c Geohealth and Hydrology Laboratory, Department of Environmental Engineering Sciences, University of Florida, Gainesville, Florida, USA; University of Michigan—Ann Arbor

**Keywords:** *Vibrio parahaemolyticus*, *Vibrio vulnificus*, Chesapeake Bay, virulence determinants, predictive intelligence, environmental microbiology, pathogens, climate change, temperature, salinity, chlorophyll

## Abstract

Incidence of vibriosis is rising globally, with evidence that changing climatic conditions are influencing environmental factors that enhance growth of pathogenic *Vibrio* spp. in aquatic ecosystems. To determine the impact of environmental factors on occurrence of pathogenic *Vibrio* spp., samples were collected in the Chesapeake Bay, Maryland, during 2009 to 2012 and 2019 to 2022. Genetic markers for Vibrio vulnificus (*vvhA*) and Vibrio parahaemolyticus (*tlh*, *tdh*, and *trh*) were enumerated by direct plating and DNA colony hybridization. Results confirmed seasonality and environmental parameters as predictors. Water temperature showed a linear correlation with *vvhA* and *tlh*, and two critical thresholds were observed, an initial increase in detectable numbers (>15°C) and a second increase when maximum counts were recorded (>25°C). Temperature and pathogenic V. parahaemolyticus (*tdh* and *trh*) were not strongly correlated; however, the evidence showed that these organisms persist in oyster and sediment at colder temperatures. Salinity (10 to 15 ppt), total chlorophyll *a* (5 to 25 μg/L), dissolved oxygen (5 to 10 mg/L), and pH (8) were associated with increased abundance of *vvhA* and *tlh*. Importantly, a long-term increase in *Vibrio* spp. numbers was observed in water samples between the two collection periods, specifically at Tangier Sound (lower bay), with the evidence suggesting an extended seasonality for these bacteria in the area. Notably, *tlh* showed a mean positive increase that was ca. 3-fold overall, with the most significant increase observed during the fall. In conclusion, vibriosis continues to be a risk in the Chesapeake Bay region. A predictive intelligence system to assist decision makers, with respect to climate and human health, is warranted.

**IMPORTANCE** The genus *Vibrio* includes pathogenic species that are naturally occurring in marine and estuarine environments globally. Routine monitoring for *Vibrio* species and environmental parameters influencing their incidence is critical to provide a warning system for the public when the risk of infection is high. In this study, occurrence of Vibrio parahaemolyticus and Vibrio vulnificus, both potential human pathogens, in Chesapeake Bay water, oysters, and sediment samples collected over a 13-year period was analyzed. The results provide a confirmation of environmental predictors for these bacteria, notably temperature, salinity, and total chlorophyll *a*, and their seasonality of occurrence. New findings refine environmental parameter thresholds of culturable *Vibrio* species and document a long-term increase in *Vibrio* populations in the Chesapeake Bay. This study provides a valuable foundation for development of predicative risk intelligence models for *Vibrio* incidence during climate change.

## INTRODUCTION

Changing climatic conditions have been linked to an increased frequency of anomalous weather events, e.g., heat waves, hurricanes, and severe precipitation, that have a severe impact on the marine environment, notably changes in sea surface height, temperature, and salinity, and for coastal communities (1). What is beginning to be understood is that environmental factors also influence incidence and transmission of pathogenic agents, i.e., proliferation, dissemination, and virulence, as well as transmission routes, notably via vectors, food, and water (2). Changes in human behavior are also associated with climate by bringing people into more frequent contact with pathogens from increased water-related activities during periods of extended warming (2, 3). In the United States, it is estimated that ca. 7.15 million waterborne illnesses occur annually (4). Climate change associated with shifts in the geographical range of microbial species, is increasingly important in the emergence and re-emergence of disease (5). A prime example is climate-driven enhancement of the incidence of pathogenic *Vibrio* (6–13).

The genus *Vibrio* comprises ecologically significant Gram-negative bacteria native to the aquatic environment and their incidence is strongly influenced by environmental parameters (6, 11, 14). *Vibrio* spp. thrive in warm water with moderate salinity (15) and are associated with aquatic invertebrates, notably crustaceans, zooplankton, and bivalves, all of which are known to impact occurrence of these bacteria in the environment (11, 16–18). Copepods, zooplankton constituting a significant component of aquatic fauna, are a major host of *Vibrio* spp., including Vibrio parahaemolyticus and Vibrio vulnificus, and are considered a vector of Vibrio cholerae (6, 18–21). Similarly, *Vibrio* spp. have been shown to concentrate in filter-feeding shellfish, especially oysters, which are often consumed raw, thereby exposing people to large doses of potentially pathogenic agents (16, 22, 23).

*Vibrio* spp. are known to cause infection in humans, with V. cholerae being the etiological agent of cholera, an acute diarrheal disease caused by consumption of food or water containing pathogenic strains of the bacterium. The seventh cholera pandemic is in progress, and the disease continues to plague the modern world, notably when climate/weather processes, microbiological parameters, and sociological determinants intersect with population vulnerabilities and loss of water, sanitation, and hygiene (WASH) infrastructure (24, 25). However, in addition to V. cholerae, V. parahaemolyticus and V. vulnificus have been proven historically significant (11, 26). V. parahaemolyticus, first described by Fujino during a 1950 shirasu food poisoning outbreak in Japan (27), is a major cause of seafood-derived foodborne gastroenteritis in the United States (28). V. vulnificus, first reported in the United States in 1976 by Hollis et al. (29), is also a common cause of foodborne illness and can cause severe extraintestinal infections, including necrotizing fasciitis and septicemia (30). The bacterium has a fatality rate among the highest of any waterborne pathogen, i.e., greater than 50% for primary septicemia. It also is responsible for ca. 95% of all waterborne and seafood-derived foodborne deaths in the United States (30, 31). In the United States, *Vibrio* spp. are estimated to cause 80,000 illnesses and hundreds of deaths annually, of which ca. 65% are foodborne (28, 32). According to the Centers for Disease Control and Prevention FoodNet, which has sites in 10 states covering 15% of the United States population, there is indication of a long-term increase in reported vibriosis between 1996 and 2019 (11, 33). In the eastern United States between 1988 and 2018, V. vulnificus wound infections increased 8-fold, i.e., from 10 to 80 cases per annum, and the northern case limit has shifted northwards 48 km per annum (13). Disturbingly, this trend of increased vibriosis is expected to continue significantly because of climate change.

Compared to clinical isolates, traditionally most V. parahaemolyticus strains collected from the environment are not considered pathogenic to humans, i.e., when primary virulence factors are absent, namely, thermostable direct hemolysin (encoded by *tdh*) and thermostable direct-related hemolysin (encoded by *trh*), which potentially carry out lysis of human erythrocytes (34–37). However, it has been estimated that up to ca. 27% of V. parahaemolyticus clinical isolates do not carry *tdh* and/or *trh* (38), suggesting that other virulence factors exist. In fact, many environmental isolates lacking *tdh* and/or *trh* can be highly cytotoxic to human gastrointestinal cells (36), with the potential to cause acute gastroenteritis (39). Furthermore, *tdh*/*trh*-negative V. parahaemolyticus strains can cause severe infections in marine fish (40) and shrimp (41), resulting in significant economic burden and aquaculture loss (42). Nonetheless, both *tdh* and *trh* are commonly used to identify pathogenic V. parahaemolyticus strains (26). An additional hemolysin, thermolabile hemolysin (*tlh*) which encodes phospholipase A2 (43), has been observed in nearly all strains of V. parahaemolyticus and is commonly employed as a marker for species detection and identification (37). In contrast, the mechanisms by which V. vulnificus causes infection in humans are more complex (37). While a few putative virulence markers have been suggested to be associated with clinical strains, e.g., pilus-type IV assembly genes (44, 45) and the virulence-correlated gene (*vcg*) (46), the extent to which they are responsible is unclear, and no single virulence gene has yet been identified that distinguishes pathogenic and nonpathogenic isolates (30). However, the V. vulnificus hemolysin A gene (*vvhA*) has been established as reliable species-specific biomarker (47). Since *Vibrio* spp. play a critical role in the degradation of polymeric substances (such as chitin), in nutrient cycling, and in other biogeochemical processes (48–51), they cannot be eradicated from the environment.

While V. cholerae and noncholera vibrios have various routes of environmental transmission, primarily attributed to the ability of V. cholerae to thrive in freshwater as well as coastal ecosystems (14) through association with a variety of living organisms, e.g., on chitinous exoskeletons of zooplankton (6, 52), treatment of vibriosis and cholera is similar and generally includes oral or intravenous rehydration therapy (28). However, antibiotics are used in prolonged and severe cases, notably wound infections caused by V. vulnificus (53). Whereas cholera has essentially been eliminated in developed countries by treatment and distribution of safe potable water, pathogenic noncholera vibrios continue to be a significant public health burden, especially following anomalous climatic events. Significant variability has been reported in their abundance and behavior among regions of individual countries and the world. Studies of environmental parameters influencing the ecological niche of pathogenic *Vibrio* strains can be helpful in developing an early warning system and risk reduction strategies.

Here, we describe analysis of environmental parameters influencing occurrence and abundance of pathogenic *Vibrio* spp. in samples collected in the Chesapeake Bay over a 13-year span, with samples collected between 2009 and 2012 and between 2019 and 2022. Specifically, we determined association of environmental parameters with abundance of V. parahaemolyticus and V. vulnificus, with the intent to improve existing models for assessing risk of infection with pathogenic *Vibrio* spp. in a changing climate.

## RESULTS

### Environmental parameters.

**FIG 1:**
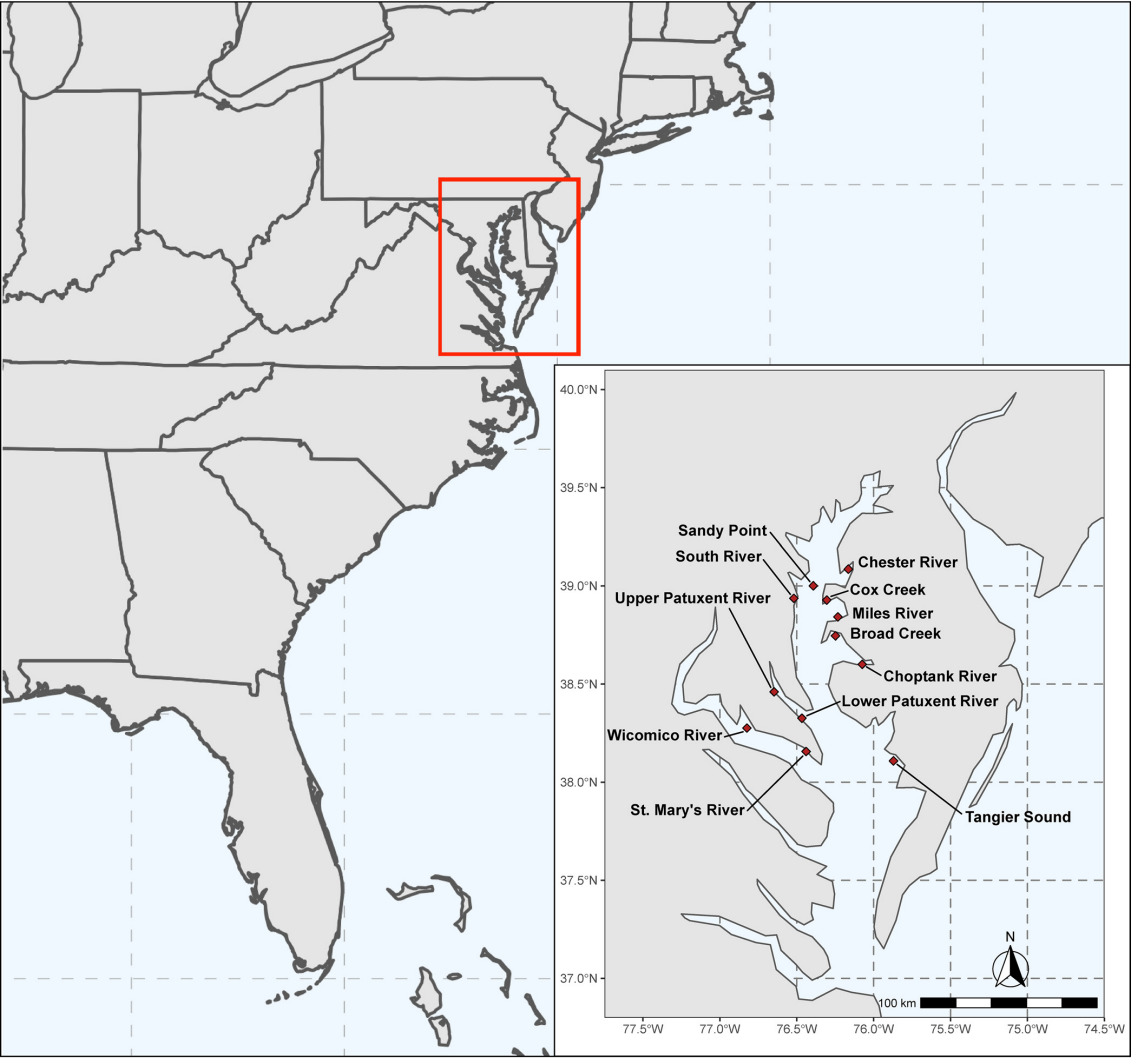
Map of sampling locations in the Chesapeake Bay. The map shows the eastern seaboard of the United States. The inset shows sampling locations, indicated by red diamonds. The scale bar corresponds to distance using World Map Data from Natural Earth (121).

**FIG 2:**
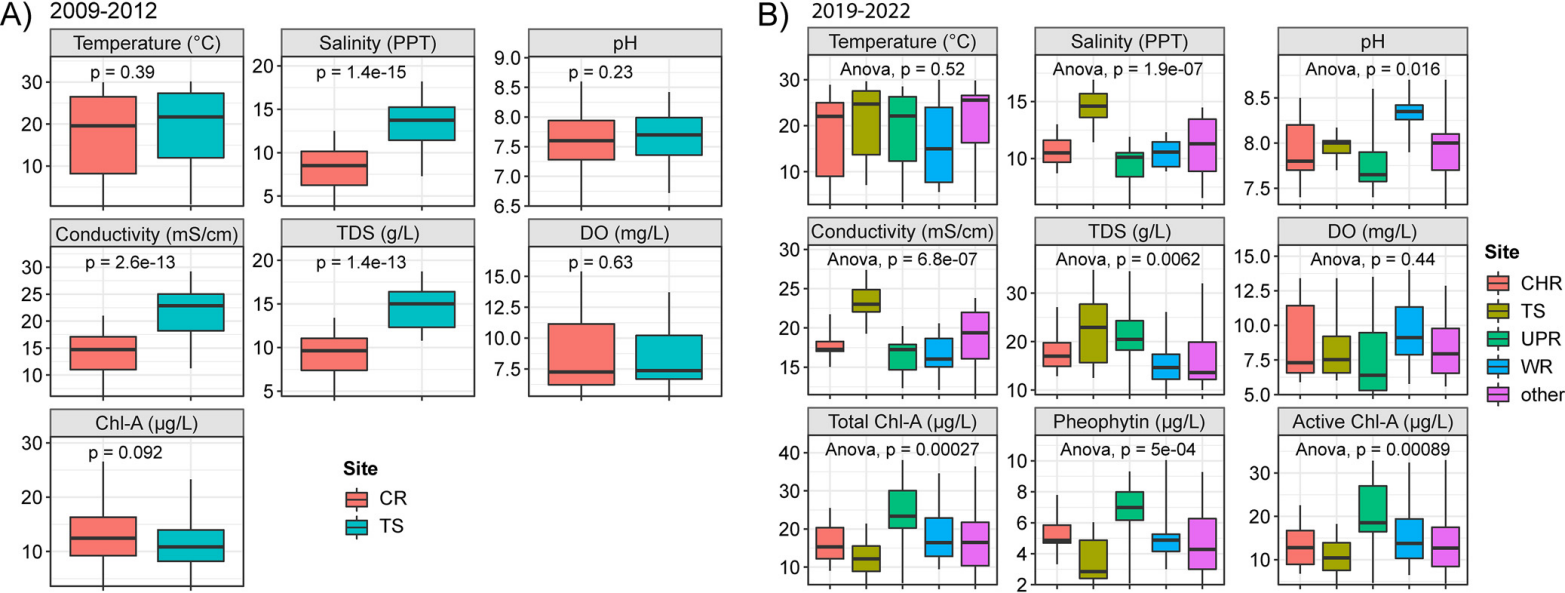
Box plots of environmental parameters. Boxes summarize distribution by indication of interquartile range (IQR), with the median shown as the center bar of each group. Whiskers represent 1.5 times the IQR. Additional circles indicate outlier values. Shown are environmental parameters for samples collected between 2009 and 2012 (A) and between 2019 and 2022 (B). CR, Chester River; TS, Tangier Sound; CHR, Choptank River; UPR, Upper Patuxent River; WR, Wicomico River.

Between June 2009 and August 2012, a total of 111 total field observations were recorded from the Chester River (CR) and Tangier Sound (TS), and between April 2019 and August 2022, 86 total field observations were recorded from 12 stations (Fig. 1). A summary of environmental parameters for each station is presented in Table 1. During all of the sampling, ranges of surface water temperature and optical dissolved oxygen (DO) were similar across the stations. However, between 2009 and 2012, differences were observed for salinity, conductivity, and total dissolved solids (TDS), namely, at TS, which showed higher values than CR (Wilcoxon, *P* < 0.05) (Fig. 2A), with the greatest differences observed during the summer and fall. Similarly, between 2019 and 2022, higher salinity, conductivity, and TDS values were recorded at TS than at other stations (analysis of variance [ANOVA], *P* < 0.05) (Fig. 2B). For total chlorophyll *a* (Chl *a*), pheophytin, and active Chl *a*, the highest values were recorded at the Upper Patuxent River (UPR) station, while the lowest values were recorded at TS (ANOVA, *P* < 0.05). For other environmental parameters, little variation was observed among the other stations, but values varied by season at each station.

**TABLE 1:** Sampling location information^*a*^

Collection period	Station^*b*^	Latitude	Longitude	Sample media^*c*^; no. of sampling events	Temp (°C)	Salinity (ppt)	pH	Conductivity (mS/cm)	Optical dissolved oxygen (mg/L)	Total Chl *a* (mg/L)^*d*^	Pheophytin (mg/L)^*d*^	Active Chl *a* (mg/L)^*d*^
2009–2012	CR	39.0859796	−76.164233	Wa, Oys, Sed; 57	0.50, 19.80, 30.20	3.2, 8.43, 12.5	5.77, 7.55, 8.8	5.79, 14.61, 21.00	5.20, 7.23, 15.39	0.06, 5.19, 41.39	ND	ND
	TS	38.1086036	−75.870907	Wa, Oys, Sed; 54	0.1, 21.48, 30.14	7.3, 13.77, 19.10	6.12, 7.70, 8.42	11.27, 15.12 18.88	5.36, 7.44, 19.5	0.03, 3.05, 10.19	ND	ND
2019–2022	BC	38.74596	−76.248124	Wa, Oys; 2	25.90, 29.20	8.80, 14.10	7.60, 8.20	16.93	6.40, 6.50	15.56, 16.48	5.27, 6.79	12.62,12.66
	CR	39.0859796	−76.164233	Wa, Oys; 1	25.2	3.72	7.32	6.82	8	21.76	9.28	16.59
	CHR	38.6006577	−76.07417	Wa, Oys; 16	2.90, 22.05, 28.90	8.70, 10.50, 13.00	7.40, 7.80, 8.50	15.03, 12.27, 21.73	5.90, 7.31, 13.40	9.04, 15.29, 25.54	3.32, 4.87, 7.79	6.78, 12.78, 22.55
	COX	38.9283561	−76.304835	Wa, Oys; 6	3.71, 16.93, 26.43	7.62, 12.05, 14.47	7.70, 7.97, 8.98	13.18, 20.39, 23.80	7.20, 9.41, 12.86	6.72, 13.88, 61.95	1.98, 4.63, 22.96	5.61, 11.29, 49.135
	LPR	38.325942	−76.466414	Wa, Oys; 7	9.30, 26.50, 28.70	10.40, 12.75, 13.80	7.40, 8.05, 8.20	17.71, 20.39, 22.73	3.85, 5.94, 11.40	7.70, 12.36, 25.07	2.66, 3.35, 6.27	6.06, 10.62, 21.55
	UPR	38.4606929	−76.646907	Wa, Oys; 17	3.40, 22.14, 28.50	5.96, 10.10, 11.90	6.60, 7.60, 8.60	10.58, 16.22, 20.22	5.00, 7.35, 14.10	5.81, 27.64, 137.82	2.09, 7.72, 29.00	4.64, 22.52, 127.52
	MR	38.842556	−76.232417	Wa, Oys; 1	16.8	14.2	8.1	23.44	9.7	12.05	2.76	10.51
	SP	39.001192	−76.392426	Wa, Oys; 1	26.08	6.54	7.51	11.51	6.11	21.78	7.72	17.48
	SR	38.937111	−76.518583	Wa, Oys; 1	30.9	6.7	8.1	11.81	7.1	74.98	8.91	69.91
	SMR	38.1565346	−76.438875	Wa, Oys; 4	3.40, 21.50, 29.80	8.00, 8.77, 9.20	8.60, 8.70, 8.90	13.70, 15.88, 20.57	7.90, 9.30, 10.71	5.66, 18.93, 36.38	2.06, 4.14, 6.02	4.51, 16.61, 32.98
	TS	38.1086036	−75.870907	Wa, Oys; 15	1.75, 24.36, 29.66	11.44, 14.96, 17.00	7.70, 8.00, 8.170	19.27, 23.05, 27.62	6.02, 7.60, 16.70	4.78, 11.60, 21.41	2.00, 2.85, 6.03	3.67, 10.21, 18.23
	WR	38.2757472	−76.824132	Wa, Oys; 15	5.60, 15.00, 29.96	5.96, 10.10, 11.90	7.90, 8.35, 8.70	5.05, 15.70, 20.58	5.00, 7.35, 14.10	9.46, 16.88, 40.59	3.00, 4.88, 10.05	6.48, 14.57, 37.64

a Results from stations with more than three sampling events are presented as minimum, median, maximum. If fewer than three sampling events were conducted at a given location, all values are shown. ND, not determined.

b BC, Broad Creek; CHR, Choptank River; CR, Chester River, COX, Cox Creek; LPR, Lower Patuxent River; MR, Miles River; SMR, St. Mary’s River; SP, Sandy Point; SR, South River; TS, Tangier Sound; UPR, Upper Patuxent River; WR, Wicomico River.

c Wa, water; Oys, oyster; Sed, sediment.

d For samples collected between 2009 and 2019, concentrations of total Chl *a* were measured in methanol extracts using methods described in references 68 and 112, performed in experimental triplicate, and the data are averages. For samples collected between 2019 and 2022, concentrations of Chl *a* (total and active) and pheophytin were measured in acetone extracts following methods described in reference 113, performed in experimental duplicate, and the data are averages.

### Distribution of V. parahaemolyticus and V. vulnificus employing DPCH.

**FIG 3:**
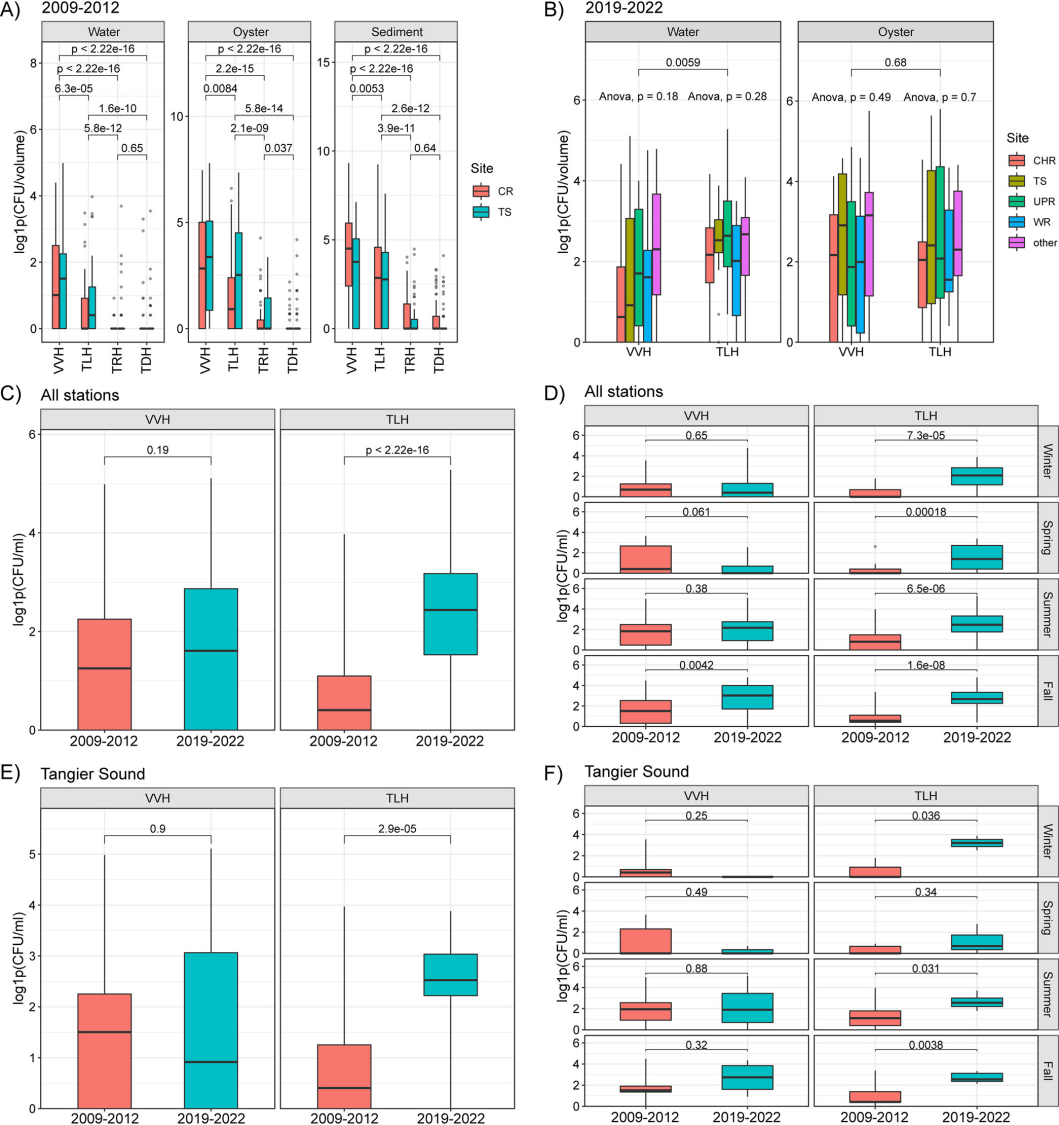
Box plots of DPCH results. Boxes summarize distribution by indication of IQR, with the median shown as the center bar of each group. Whiskers represent 1.5 times the IQR. Additional circles indicate outlier values. (A and B) DPCH results for samples collected between 2009 and 2012 (A) and between 2009 and 2012 (B). (C to F) Comparison of collection periods at all stations (C), all stations by season (D), Tangier Sound (E), and Tangier Sound by season (F).

Direct plating and DNA colony hybridization (DPCH) was used to determine incidence and abundance of V. parahaemolyticus and V. vulnificus in various sample types (Fig. 3). Overall, DPCH detection rates varied significantly by season and were highest in sediment samples, followed by oyster and water. Between 2009 and 2012, the abundance of *vvhA* was similar between TS and CR for all sample types, but *tlh* was detected at a higher abundance at the TS station for water and oyster samples (Wilcoxon, *P* < 0.05) (Fig. 3A). Overall, *trh* and *tdh* were detected at a similar abundance at both stations. No differences between stations were observed for sediment samples, among all genetic markers tested. The *vvhA* marker was detected at a higher abundance than *tlh* for all sample types (Wilcoxon, *P* < 0.05). Both *vvhA* and *tlh* were detected at a higher abundance than *trh* and *tdh* (Wilcoxon, *P* < 0.05). Between 2019 and 2022, concentrations of *tlh* and *vvhA* showed no overall difference with respect to sampling station (ANOVA, *P* > 0.05) (Fig. 3B), and *tlh* abundance was similar to *vvhA* abundance in water and oyster samples (Wilcoxon, *P* > 0.05).

### Temporal evaluation of V. parahaemolyticus and V. vulnificus.

To evaluate how concentrations of V. parahaemolyticus and V. vulnificus may have changed over time, DPCH results for water samples were compared between the two collection periods. Across all stations, no overall difference was observed between the two collection periods for *vvhA*, but *tlh* concentrations increased significantly during the 2019–2022 collection period compared to 2009 to 2012, whereby median log1p, i.e., [log(*x* + 1)], values increased from 0.4 to 2.4 (Wilcoxon, *P* < 0.05) and mean positive values increased from ca. 5 CFU/mL to ca. 18 CFU/mL (Fig. 3C). With respect to season, an increase in *vvhA* was observed during the fall, where median log1p values increased from 1.5 to 3 (Wilcoxon, *P* < 0.05) and mean positive values increased from ca. 18 CFU/mL to ca. 37 CFU/mL (Fig. 3D). Similarly, *tlh* showed significant increases during all seasons (Wilcoxon, *P* < 0.05), with the greatest increase observed during the fall, when median log1p values increased from 0.55 to 2.67 and mean positive values increased from ca. 3 CFU/mL to ca. 22 CFU/mL.

Further evaluation of water samples at TS allowed for temporal evaluation of *vvhA* and *tlh* from the same station between collection periods. Water samples from the TS station showed no overall difference for environmental parameters, including temperature, salinity, conductivity, TDS, DO, and total Chl *a*, between the two periods (Wilcoxon, *P* > 0.05). Like the comparison of all stations, no statistical difference was observed for the concentration of *vvhA*, but an increase was observed for *tlh*, in which median log1p values increased from 0.41 to 2.43 (Wilcoxon, *P* < 0.05) and mean positive values increased from ca. 5 CFU/mL to ca. 18 CFU/mL (Fig. 3E). With respect to seasonality, *vvhA* did not show a significant difference, but *tlh* showed significant differences during the summer, fall, and winter (Wilcoxon, *P* < 0.05), with the greatest increase observed during the fall, when median log1p values increased from 0.55 to 2.67 and mean positive values increased from ca. 2 CFU/mL to ca. 22 CFU/mL (Fig. 3F).

### Seasonal impact on incidence of pathogenic *Vibrio*.

**FIG 4:**
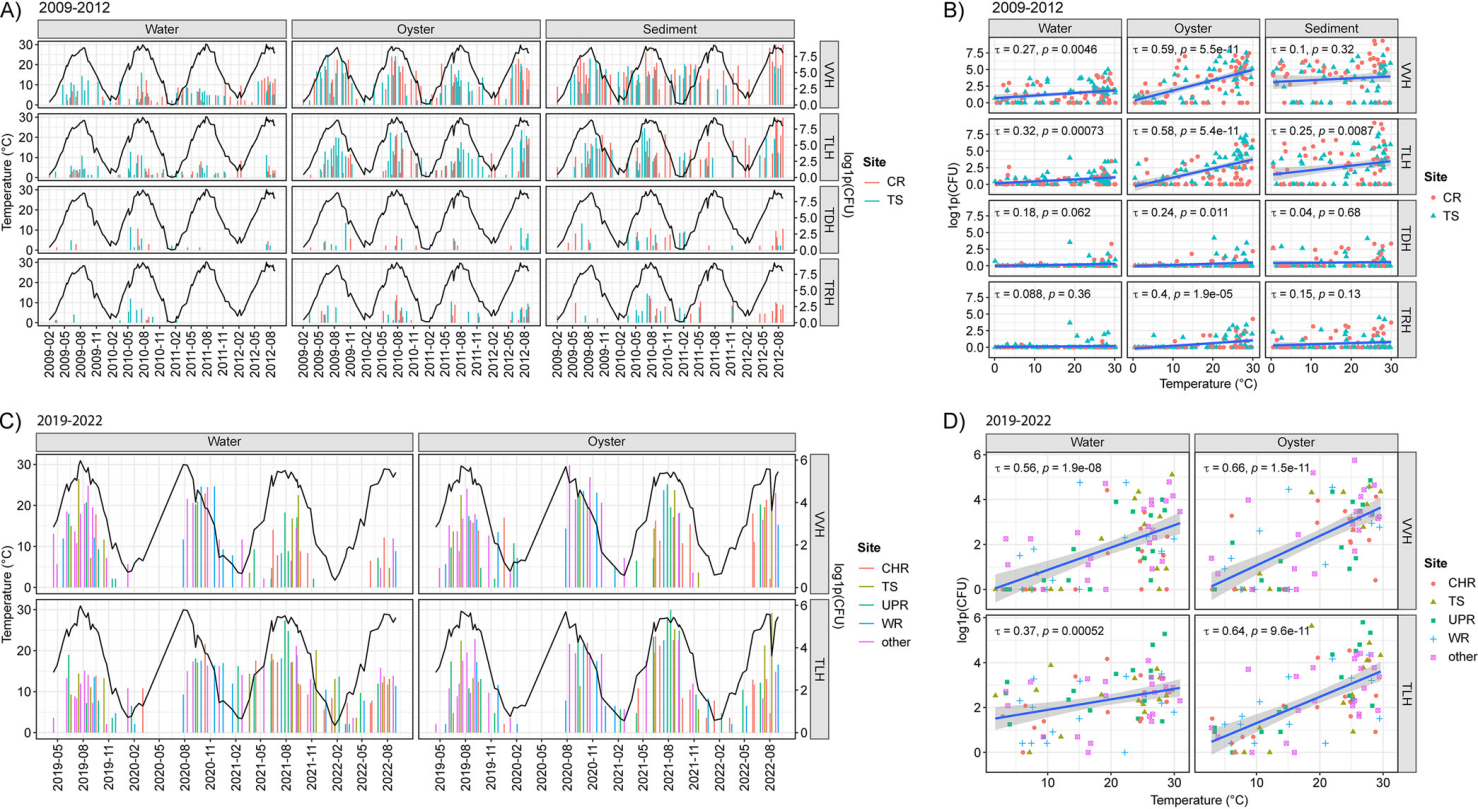
Seasonal association of DPCH results. Bar plots show DPCH results on the right axis and temperature values (black lines) on the left. Scatterplots include linear regression, with 95% confidence intervals represented by shaded regions. Correlations among axis variables were respectively generated using Kendall’s tau method. Shown are DPCH results and temperature for samples collected between 2009 and 2012 (A and B) and 2019 to 2022 (C and D).

To assess the impact of seasonality, the DPCH results were evaluated with respect to water temperature at the time of sample collection (Fig. 4). For each sample type and location, the range of DPCH detection and percentage of positive samples relative to the total number are shown in Table 2. The number of *tlh* and *vvhA* determinations showed strong linear association with temperature, with the highest abundance occurring in the warmer months of the year. For samples collected between 2009 and 2012 (Fig. 4A), the greatest association with temperature was observed in oyster samples for both *vvhA* and *tlh* (tau > 0.5) (Fig. 4B). However, it is worth noting that *vvhA* and occasionally *tlh* were detected in higher numbers during the winter months in sediment samples collected from both CR and TS stations. Hence, the correlation between temperature and *vvhA* was low for sediment samples (tau < 0.1). Compared to *tlh*, negative results were more frequent when DPCH was used to quantitate *trh* and *tdh* populations in water, oyster, and sediment samples from both stations between 2009 and 2012. Both *tdh* and *trh* were detected during warmer months, with higher detection in oyster and sediment samples. As for *tlh* and *vvhA*, the *trh* and *tdh* detection rates were highest for sediment, followed by oyster and water. Pathogenic V. parahaemolyticus (*trh* and *tdh*) were detected more frequently during the winter months in sediment than in water and oysters. With exception of *trh* in oysters (tau = 0.4), the correlation with temperature was not strong. A similar pattern of seasonality was observed for samples collected between 2019 and 2022 (Fig. 4C), where *tlh* and *vvhA* showed a stronger association with temperature for oyster samples (*tlh*, tau = 0.64; *vvhA*, tau = 0.66) than water samples (*tlh*, tau = 0.37; *vvhA*, tau = 0.56) (Fig. 4D).

**TABLE 2:** Summary for Vibrio parahaemolyticus and Vibrio vulnificus detection employing DPCH

Collection period	Site	DPCH result for^*a*^:											
		Water (CFU/mL)				Oyster^*b*^				Sediment (CFU/g)			
		*vvhA*	*tlh*	*trh*	*tdh*	*vvhA*	*tlh*	*trh*	*tdh*	*vvhA*	*tlh*	*trh*	*tdh*
2009–2012	CR	80 (3.5) [0.69]	31.5 (1.5) [0.46]	1.5 (<1) [0.07]	26 (1.5) [0.12]	1.73 × 10^4^ (515) [0.71]	7.4 × 10^3^ (72.5) [0.57]	705 (25) [0.27]	80 (12.5) [0.18]	1.12 × 10^5^ (1.87 × 10^3^) [0.74]	1.03 × 10^5^ (465) [0.65]	550 (110) [0.30]	270 (75) [0.29]
	TS	145 (6) [0.70]	52 (1.5) [0.69]	39 (2) [0.19]	33.5 (1) [0.17]	2.43 × 10^4^ (440) [0.81]	1.56 × 10^4^ (535) [0.74]	280 (50) [0.35]	650 (40) [0.22]	1.24 × 10^4^ (105) [0.70]	2.00 × 10^5^ (45) [0.62]	870 (5) [0.26]	600 (60) [0.24]
2019–2022	BC	119, 15.5 [1]	58.5, 36.25 [1]			310, 225 [1]	770, 55 [1]						
	CR	34.5 [1]	12 [1]			175 [1]	70 [1]						
	CHR	82 (7) [0.5]	63.5 (8.5) [0.94]			610 (185) [0.69]	925 (70) [0.94]						
	COX	111 (27) [1]	27.5 (14.5) [1]			810 (402.5) [0.83]	1.79 × 10^3^ (525) [1]						
	LPA	50.5 (2.5) [0.71]	26 (17.5) [1]			1.03 × 10^3^ (265) [0.71]	770 (225) [0.86]						
	UPA	53.5 (5) [0.75]	195 (13.75) [1]			3.28 × 10^4^ (95) [0.75]	1.28 × 10^4^ (172.5) [0.94]						
	MR	3 [1]	<1 [1]			105 [1]	55 [1]						
	SP	7 [1]	4 [1]			45 [1]	90 [1]						
	SR	31 [1]	9 [1]			245 [1]	280 [1]						
	SMR	63.5 (10.75) [1]	17 (14) [0.75]			3145 (80) [1]	640 (45) [0.75]						
	TS	164.5 (12.75) [0.67]	47.5 (11.75) [0.93]			960 (430) [0.75]	2.78 × 10^3^ (425) [0.75]						
	WR	115.5 (6.75) [0.67]	32 (7.25) [0.93]			965 (165) [0.71]	76 (37.5) [1]						

a Results from stations with more than three sampling events are presented as maximum (median of positive samples). If fewer than three sampling events were conducted at a given location, all values are shown. Values in brackets are proportions of positive samples relative to total number of samples. All assays were performed in duplicate, and the data are averages, prior to statistical evaluation.

b For samples collected between 2009 and 2012, values are CFU per gram; for samples collected between 2019 and 2022, values are CFU per 100 μL.

### Environmental parameters associated with incidence.

**FIG 5:**
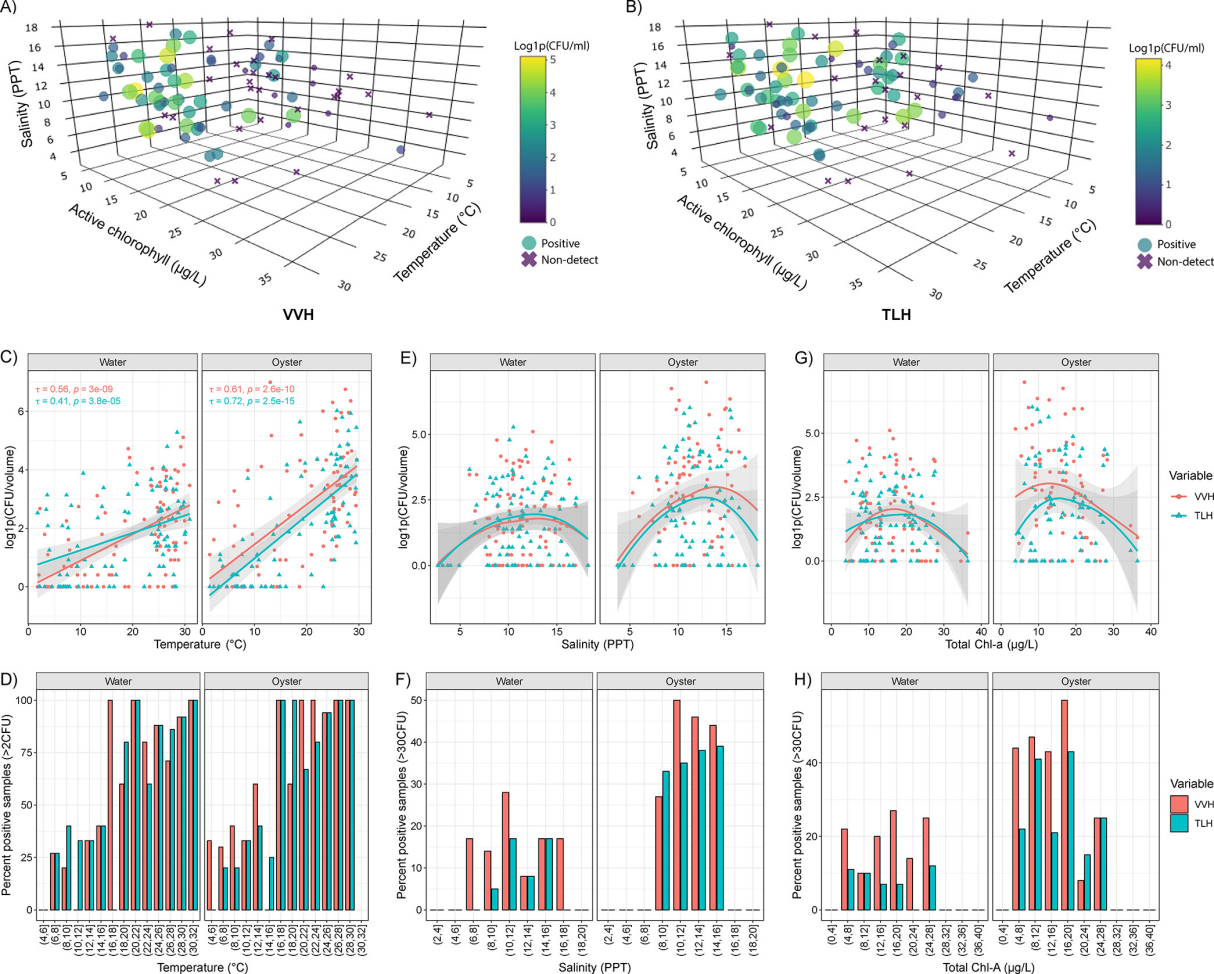
Association of temperature, salinity, and total chlorophyll *a* with occurrence of Vibrio vulnificus and Vibrio parahaemolyticus. Three-dimensional scatterplots are shown for temperature, salinity, and total chlorophyll *a*; marker size and color are indicative of detection and abundance of *vvhA* (A) and *tlh* (B). Two-dimensional scatterplots show the association of environmental parameters with direct plating and DNA colony hybridization results and include linear regression, with 95% confidence intervals represented by shaded regions. Correlations among axis variables for temperature and DPCH results were generated using Kendall’s tau method. A nonparametric regression model, i.e., LOESS, was used to visualize the relationship of DPCH results with environmental parameters lacking linear correlation, i.e., salinity and total chlorophyll *a*. Bar plots represent the percent positive samples above defined hybridization thresholds, following the binning of environmental parameter values. Two-dimensional scatterplots and bar plots are shown for temperature (C and D), salinity (E and F), and total chlorophyll *a* (G and H).

The relationship among environmental parameters and DPCH results was further investigated to determine optimal ranges of each environmental parameter associated with genetic marker abundance. Figure 5 shows analysis of pooled data for both collection periods. Temperature, salinity, and total Chl *a* were determined to be strong predictors of increased abundance of both *vvhA* (Fig. 5A) and *tlh* (Fig. 5B), forming distinct clusters compared to sampling events with low abundance or those for which genetic markers were not detected. Analysis of individual stations, shown as scatterplots and overlaid kernel density of each genetic marker detected above the threshold of 10 CFU per hybridization, can be found in Fig. S1 in the supplemental material.

### (i) Temperature.

With respect to seasonal temperature, occurrence of both *tlh* and *vvhA* increased proportionally with temperature during both collection periods (tau > 0.4), with the greatest association observed for *vvhA* occurrence in oyster samples (Fig. 5C). As temperature increased, the percent of positive samples also increased, and the temperature threshold for occurrence of *tlh* and *vvhA* in >50% of the samples was determined to be >15°C (Fig. 5D). A second temperature range, indicative of increased to maximum marker abundance, was observed when temperatures were >25°C. However, it should be noted that in a few cases increased *tlh* and *vvhA* numbers were observed in oyster and sediment samples at lower temperatures during the 2009–2012 collection period (Fig. S1). Incidence of both *trh* and *tdh* was associated with temperatures of >20°C in oyster and water samples, but moderate abundance of these markers was detected occasionally at lower temperatures in sediment samples compared to water and oyster samples.

### (ii) Salinity.

The overall relationship between salinity and abundance of both *tlh* and *vvhA* was nonlinear, demonstrative of a downward curve (Fig. 5E). Evaluation of pooled data showed salinity ranges associated with positive samples above the threshold of 30 CFU/hybridization to be between 6 ppt and 18 ppt for *vvhA* and 8 ppt and 16 ppt for *tlh* (Fig. 5F). However, a salinity range between 10 ppt and 15 ppt was associated with increased to maximum marker abundance. For samples collected between 2009 and 2012, the optimal salinity ranges for *tlh* and *vvhA* differed between CR and TS stations, where the salinity of CR was lower than TS (Fig. S1). Moderate abundance of *vvhA* was associated with salinity ranges between 3 ppt and 12.5 ppt at CR and ranges between 10 ppt and 17 ppt at TS. Differences in salinity between stations were more prominent for *tlh* in water (CR, 3 to 8 ppt; TS, 12 to 16 ppt) than in oyster and sediment (CR, 4 to 15 ppt; TS, 10 to 17 ppt). For samples collected between 2019 and 2022, salinity ranges associated with moderate abundance were more similar among sampling stations, with exception of *tlh* detection in water at Choptank River (CHR) and UPR stations (6 to 12 ppt) compared to TS (12 to 17 ppt), whereby salinity ranges associated with moderate abundance were observed between 7 and 17 ppt for both *tlh* and *vvhA* (Fig. S1).

### (iii) Chlorophyll and pheophytin.

As for salinity, the relationship of total Chl *a* and detection of *tlh* and *vvhA* was observed as a nonlinear downward curve (Fig. 5G). Across both collection periods, total Chl *a* values associated with an increased number of positive samples with >30 CFU/hybridization for both *vvhA* and *tlh* were determined to be between 4 μg/L and 28 μg/L (Fig. 5H). However, higher values were observed, ranging from 20 μg/L to 40 μg/L at the UPR station (Fig. S1). For *trh* and *tdh*, a peak density was observed around 10 μg/L, but the overall range of detection was like that of *tlh* and *vvhA*. For samples collected between 2019 and 2022, optimal ranges for pheophytin (4 to 8 μg/L) and active Chl *a* (10 to 20 μg/L) were observed to be associated with *tlh* and *vvhA* detection at increased abundance.

### (iv) Other environmental parameters.

The relationship of other environmental parameters and *Vibrio* abundance was consistent across collection periods and location, observed as a nonlinear relationship with an acceptable range for occurrence of genetic markers. For all markers, pH ranges between 7 and 9 were associated with moderate detection, and pH values closer to 8 were generally associated increased abundance (Fig. S1). DO ranges between 4 mg/L and 15 mg/L were associated with moderate detection of *vvhA* and *tlh*. The DO range for increased abundance of *tlh* and *vvhA* was observed between 5 mg/L and 8 mg/L, which was also within the range for presence of pathogenic V. parahaemolyticus markers. Ranges for conductivity (15 to 22 mg/L) were associated with increased abundance of *vvhA* and *tlh* during both periods. Between 2009 and 2012, TDS ranges (10 to 15 g/L) were associated with increased abundance, while slightly higher values were observed (15 to 25 g/L) during the 2019–2022 collection period.

## DISCUSSION

Climate change is recognized to be associated with increased frequency of anomalous weather events, which can result in warmer and less saline waters along the coast (1). In general, *Vibrio* spp. thrive in warm water and moderate salinity (11, 15). An increasing number of studies document a pattern of poleward spreading of noncholera *Vibrio* spp., demonstrating significant geographic expansion of *Vibrio* spp. populations (6, 7, 9, 10, 12, 54, 55), corroborated by associated impacts on human health—especially in coastal communities, including the eastern seaboard of the United States (13) and the Chesapeake Bay (56, 57). Unfortunately, the number of cases of vibriosis is expected to increase further in coming years as a result of climate variabilities, notably changes in frequency and magnitude of anomalous weather events, e.g., heat waves, hurricanes, floods, and droughts (6, 9, 11, 54, 58–60). A prime example is the number of V. parahaemolyticus and V. vulnificus cases reported along coastal regions of the Florida Gulf following Hurricane Ian, a destructive category 4 Atlantic hurricane that made landfall on Florida in September 2022. There were 74 confirmed cases of V. vulnificus infection and 17 deaths reported that year—nearly double the normal number for the area (61). It is reasonable to suspect that changes in frequency, intensity, and duration of extreme weather events will affect the ecology of pathogenic *Vibrio* spp., notably in the Chesapeake Bay, which is already experiencing major climate-driven changes, namely, sea level rise and flooding associated with storms and hurricanes (56). Hence, it is important to understand the ecology of *Vibrio* spp. and environmental parameters influencing their occurrence and transmission in a changing climate.

A primary objective of the research reported here was to determine microbial ecological niche profiles that can be incorporated in predictive risk assessment models and real-time remote sensing data for geographic regions of the world. Risk prediction models provide an early warning, essential to safeguarding public health, especially in regions vulnerable to infrastructure instability (e.g., lack of WASH infrastructure), natural calamity (e.g., hurricanes, floods, and earthquakes), and/or social disruption and civil unrest (e.g., wars, coups, political crises, and economic recessions). For example, the risk of cholera can now be predicted at the scale of available satellite observation and favorable environmental conditions (62, 63). The model was employed to assess the risk of cholera in Ukraine (24) and Yemen (64), countries suffering damaged or severely crippled civil infrastructure, following which the human population is at risk of health disasters. It is important to note that both cholera and vibriosis have various routes of environmental transmission, primarily attributed to the ability of V. cholerae to thrive in freshwater through its salinity requirements (14) and association with a variety of hosts with chitinous exoskeletons (6, 52). Other models, such as the National Centers for Coastal Ocean Science (NCCOS) Probability Model for V. vulnificus Occurrence in Chesapeake Bay Water (65) and the *Vibrio* Map Viewer (66) developed by the European Center for Disease Prevention and Control (ECDC), have been developed to predict increased incidence of *Vibrio* spp. in the environment. Data from this study will be used to refine the established models by providing additional environmental parameter thresholds, including temperature, salinity, and chlorophyll, for V. parahaemolyticus and V. vulnificus. Design of future predictive risk assessment models for *Vibrio* spp. will benefit from inclusion of sociological and behavioral factors related to contact with the aquatic environment, such as swimming, fishing, and other recreational/occupational activities, that influence contact with waterborne pathogenic agents.

Over the past 5 decades, extensive data sets have been generated with respect to incidence and behavior of V. cholerae and noncholera *Vibrio* spp. in the ocean and estuarine environments through cross-sectional studies, including long-term retrospective work (6, 9, 18, 55, 67–75). These studies, reviewed by Brumfield et al. (11), provide evidence for environmental parameters as drivers, both directly and indirectly, of the incidence of pathogenic *Vibrio* spp. in the environment.

The results presented here represent one of the most intensive sampling campaigns for *Vibrio* spp. occurrence and distribution, allowing determination of seasonal variation of *Vibrio* abundance. However, it should be noted that *Vibrio* spp. enter the viable but nonculturable (VBNC) state (76–78), in which the cells become metabolically dormant. VBNC cells cannot be cultured employing standard laboratory media, but they are detectable using molecular genetic methods (79). V. parahaemolyticus and V. vulnificus have been shown to have increased resistance to thermal stress, low salinity, and acidic inactivation when in the VBNC state, suggestive of a strategy for survival under adverse conditions (80, 81). Interestingly, studies have shown alternative methods, such as PCR, quantitative PCR (qPCR), loop-mediated isothermal amplification (LAMP), and DNA sequencing, to be more sensitive for detection of genetic markers than DPCH (65, 68, 82–84). Additionally, improved recovery of pathogenic V. parahaemolyticus by DPCH has been observed following enrichment (85). The results reported here were for culturable *Vibrio* detected using DPCH without enrichment, hence likely underestimating total *Vibrio* populations. Alternative methods will be helpful in the future for more precise quantification of *Vibrio* spp. Complementary research is under way employing whole-genome shotgun sequencing of archived *Vibrio* spp. isolates to determine the influence of environmental factors on genomic diversity, including virulence and antimicrobial resistance.

Genetic markers of V. parahaemolyticus and V. vulnificus in various sample matrices were quantified using DPCH (Fig. 3), and the results support findings of previous studies using similar culture-based methods in estuarine waters of Louisiana, Maryland, Mississippi, Georgia, South Carolina, and Washington (USA) (68, 82, 86–89). These studies and related work in the northern Gulf of Mexico (90), conducted approximately at the same time, showed relatively few *tdh*/*trh*-positive samples. Here, we detected pathogenic V. parahaemolyticus in ca. one-third of oyster and sediment samples and ca. 14% of water samples collected between 2009 and 2012 (Table 2). The higher rates of detection of pathogenic V. parahaemolyticus in oyster and sediment samples suggest a niche of pathogenic vibrios, i.e., increased abundance of *trh*/*tdh* bacteria at temperatures of <10°C, but a lack of linear correlation with temperature. These observations support previous work showing the preferred niche of pathogenic *Vibrio* spp. in matrices other than water, especially at lower temperatures (91, 92). An *in situ* study conducted in the Mediterranean Sea showed culturable *Vibrio* spp. to be present year-round in sediment (93). It can be concluded that oysters and sediment function as reservoirs of pathogenic *Vibrio* spp. and represent different communities than those in water, contributing to seasonality of *Vibrio* spp. during unfavorable conditions.

Results of this study reaffirm temperature as a strong predictor of the incidence and distribution of *Vibrio* spp. (measured using *tlh* and *vvhA*) in the environment (6, 12, 55, 65, 68, 75, 94). A similar study of culturable V. parahaemolyticus populations in the Chesapeake Bay between 1970 and 1971 (95) found V. parahaemolyticus numbers below detectable levels in the water column from January to early April until water temperatures rose above 14°C. However, a rapid increase in counts from 0 to 10^3^ CFU/100 mL were found in the period from April to early June, and maximum counts (6.2 × 10^3^ CFU/100 mL) were observed in the middle of July. Subsequently, V. parahaemolyticus numbers stayed around 10^3^ CFU/100 mL until the middle of October when the water temperature dropped below 15°C (95). A similar pattern of seasonality was observed here (Fig. 4), whereby two critical temperature thresholds were observed at 15°C and 25°C. That is, between 14°C and 15°C, an initial increase in *Vibrio* density was observed, and when temperatures rose above 25°C, maximum counts were observed (Fig. 5).

While critical temperature thresholds have been fairly consistent over the years, the presumptive V. parahaemolyticus populations demonstrate a long-term seasonal increase between 2019 and 2022, compared to similar locations in the 1970s (86, 87, 95, 96). Maximum counts of V. parahaemolyticus in water samples during summer months were within the same order of magnitude, i.e., ~10^3^ CFU/100 mL, but detectable values were more frequent during winter and early spring, with the lowest numbers recorded as late as March. During 2019 to 2022, the numbers remained high to the end of fall. Samples collected between 2019 and 2022, showed that mean *tlh* numbers significantly increased across all seasons (ca. 3-fold) for 2019 to 2022 compared to 2009 to 2012, with the greatest increase observed during the fall for both *tlh* (ca. 7-fold) and *vvhA* (ca. 2-fold). Similarly, water samples collected at TS, the station consistently sampled during both the 2009–2012 and 2019–2022 collection periods, showed increases in *tlh* during summer, fall, and winter, with the greatest increase in the fall. However, no significant difference was observed for *vvhA* at TS between collection periods, which might be due to the fact that V. vulnificus has been found to be concentrated in the middle bay, upper bay, and western estuaries compared to stations in the lower bay (97). Hence, additional sampling from more diverse locations is required to elucidate changes in V. vulnificus populations over time. Nonetheless, *Vibrio* spp. exhibit seasonal patterns of growth, and these results suggest an extended seasonality, notably during the fall.

The methods for processing oyster samples differed between collection periods, i.e., weight (2009 to 2012) compared to volume (2019 to 2022). Thus, a direct quantitative comparison could not be made. However, the rates of detection using genetic markers were similar; e.g., the study reported in reference 82 employed DPCH during 2004 to 2005 and detected *tlh* in ca. 80% of oyster samples. In this study, ca. 70% (2009 to 2012) and ca. 85% (2019 to 2022) of the oyster samples were positive for *tlh*. Values for oyster samples evaluated by weight during 2009 to 2012 were low, according to United States Food and Drug Administration (FDA) guidance for V. parahaemolyticus (<10^4^ CFU/g) in ready-to-eat foods (98). Overall, *tlh* detection rates were about 10% lower for water than oysters, and for all genetic markers, DPCH detection rates were highest in sediment, followed by oyster and water, in agreement with previous reports (68).

Salinity proved to be a strong predictor of *Vibrio* abundance, in agreement with findings of previous studies (65, 99–102). Overall, the relationship between *Vibrio* abundance and salinity indicated a nonlinear downward curve (Fig. 5), with maximum numbers observed when the salinity was between 10 and 15 ppt, in agreement with reference 65 for V. vulnificus and an optimal salinity gradient of 11.5 ppt.

Chlorophyll serves as an important predictor of *Vibrio* abundance (103–107), especially since chlorophyll indicates density of phytoplankton populations and thus serves as an indicator of zooplankton, which feed on phytoplankton (6, 108). Since zooplankton feed on phytoplankton, correlation of zooplankton population surges following phytoplankton blooms can be calculated. *Vibrio* spp. are commonly associated with zooplankton, primarily copepods, whereby they provide nutrient-rich surfaces to which the bacteria attach (18, 69). Abundance of phytoplankton, followed by a zooplankton bloom, can be used to predict increase in the *Vibrio* populations in nutrient-rich waters (103). Phytoplankton blooms are also an indicator of nutrient runoff introducing organic matter, leading to proliferation of *Vibrio* spp. (109, 110). Here, the relationship between markers (*tlh* and *vvhA*) and chlorophyll was found to be nonlinear (Fig. 5), with an acceptable range of increased occurrence of genetic markers according to chlorophyll pigments, including total Chl *a* (5 to 25 μg/L), namely, pheophytin (4 to 8 μg/L) and active Chl *a* (10 to 20 μg/L). With respect to pathogenic V. parahaemolyticus (*trh* and *tdh*), a peak density was observed around 10 μg/L. However, chlorophyll as a proxy for zooplankton abundance would benefit by including specific lag times, e.g., 1 month, to account for a subsequent increase in zooplankton (6, 108, 111).

### Conclusions.

With a high growth rate and rapid response to environmental signals, *Vibrio* spp. have been proposed as a microbial indicator of a changing global climate, corroborated by impact on public health, namely, increased number of infections caused by these pathogens (6, 9–13, 16, 17, 19, 54, 58–60, 86, 87). Hence, it is important to understand the ecology of *Vibrio* spp. and the environmental parameters influencing their occurrence and transmission. This study, employing field sampling in the Chesapeake Bay, confirmed results of earlier studies, with respect to numbers of pathogenic *Vibrio* spp. and their correlation with environmental factors, including seasonality, temperature, salinity, and chlorophyll. In addition, correlative differences between response of total and pathogenic V. parahaemolyticus and V. vulnificus to environmental parameters were determined. It is concluded that pathogenic *Vibrio* spp. cannot be treated as a single cohesive unit, since subspecies/strains may inhabit differential niches. Further, different population composition was observed for oyster and sediment compared to water and linked to *Vibrio* seasonality. A long-term increase in *Vibrio* populations in the Chesapeake Bay was determined, particularly during fall months of the year, suggesting that an extended seasonality of these bacteria has occurred. These results provide a baseline useful for future investigations, both in the Chesapeake Bay and globally, to evaluate changes in pathogenic *Vibrio* populations relative to climate change over time. The results obtained to date will be used in developing environmental and climate niche models for noncholera *Vibrio* spp., namely, V. parahaemolyticus and V. vulnificus.

## MATERIALS AND METHODS

### Site description.

Methods employed for sample collection and processing have been described previously in detail (67); a summary of methods relative to this study is provided here. Samples were collected from the Chesapeake Bay, Maryland, USA, during two separate 3-year sampling events spanning 13 years. Between June 2009 and August 2012, water, oyster (eastern oyster [Crassostrea virginica]), and sediment samples were collected at two stations, i.e., CR and TS, totaling 111 sampling events. The Chesapeake Bay, an inlet of water enclosed on three sides by land, is the largest estuary in the United States. CR is a major tributary in the northern portion of the Chesapeake Bay comprising brackish water, while TS is an inlet of the larger Atlantic Ocean with a more saline water content and is located further south. Between April 2019 and August 2022, water and oyster samples were collected from 12 stations throughout the Chesapeake Bay, totaling 85 sampling events (Fig. 1). Details of each sampling station location, number of sampling events and samples collected are detailed in Table 1.

### Sample collection.

Between 2009 and 2012, sampling (water, oyster, and sediment) was done twice per month during warmer months (June through August) and once per month the rest of the year. Between 2019 and 2022, sampling (water and oyster) was done weekly during the warmer months and twice per month during the rest of the year. However, between March 2020 and July 2020, no samples were collected due to laboratory restrictions associated with coronavirus disease 2019 (COVID-19). Water (12 L) was collected 0.3 m below the surface using a Van Dorn water sampler (WildCo, Buffalo, NY) and stored in a sterile Nalgene carboy (Thermo Fisher Scientific, Waltham, MA, USA). The carboy was rinsed three times with sample water from the site prior to collection. Up to 30 oysters were collected by dredging and stored in clean double-zipper freezer bags. Sediment (ca. 100 g) was collected using a stainless-steel scoop and stored in sterile Nalgene bottles (Thermo Fisher Scientific, Waltham, MA, USA). Samples were transported to the laboratory in a cooler with ice packs, ensuring that samples did not come in direct contact with the ice packs. Temperature of the coolers was monitored to ensure that it did not reach above 8°C during transport (<4 h) using a LogTag single-trip temperature alert indicator (LogTag Recorders, Auckland, New Zealand). The temperature of the water samples was recorded upon arrival at the laboratory, and samples were stored at 4°C until processing (<12 h). It is worth noting that internal analysis of split samples processed immediately (i.e., no refrigeration) and following refrigeration for 12 h showed no significant change in the number of culturable bacteria detected in homogenized oyster tissue or water samples at a volume of >10 L, using methods detailed below.

### Environmental parameters.

During each sampling event, water temperature, pH, DO, salinity, conductivity, and TDS were measured 0.3 m below the surface and 0.3 m above the bottom using a handheld water probe (Eureka, Austin, TX). Air temperature was recorded at the time of collection. Total Chl *a* was measured by high-performance liquid chromatography. Briefly, volumes up to 250 mL of water were filtered using 25-mm glass microfiber filters (Whatman GF/F; Cytiva, Marlborough, MA) and stored at −80°C. For samples collected between 2009 and 2012, concentrations of total Chl *a* were measured in methanol extracts on a Cary model 50 UV–visible-light spectrophotometer, as described previously (68, 112). For samples collected between 2019 and 2022, concentrations of Chl *a* (total and active) and pheophytin were measured in acetone extracts on a Shimadzu UV 2401PC spectrophotometer following University of Maryland Center for Environmental Science (UMCES) standard operating procedures for fluorometric determination of Chl *a* in waters and sediments of fresh/estuarine/coastal areas (113), modified from United States Environmental Protection Agency (EPA) method no. 445.0 (114) and American Public Health Association (APHA) standard method no. 10200H.3 (115). Chl *a* analysis for 2009 to 2012 and 2019 to 2022 was performed in experimental triplicate and duplicate, respectively, and averages are presented.

### Sample processing.

Details of sample processing have been reported in other publications (67, 68). Briefly, samples were treated following methods outlined in the *Bacteriological Analytical Manual* for food sampling/preparation of sample homogenate (116) and *Vibrio* (117). Notably, water samples were shaken vigorously 25 times in a 30-cm arc for 7 s. Oysters were rinsed and scrubbed under running deionized water to remove debris from the shell and opened using a sterile shucking knife. Oyster tissue in an equal amount of phosphate-buffered saline (PBS; pH 7.4) was homogenized in a sterile blender for 90 s. Sediment samples were weighed in a sterile conical tube and vortexed in an equal amount of PBS for 90 s. Following homogenization, all dilutions were done in appropriate media within 15 min.

### Direct plating and DNA colony hybridization.

Methods to enumerate V. parahaemolyticus and V. vulnificus populations via DPCH have been described previously (68, 82, 90, 117). For samples collected between 2009 and 2012, water (1 mL), oyster (0.1 g or 0.01 g), and various sediment concentrations (up to 0.05 g [wet weight]) were used for DPCH. For 2019–2022 samples, water (1 mL) and oyster (100 μL of oyster homogenate) were used. Respective sample volumes were spread plated in duplicate on T1N3 agar (1% tryptone, 3% NaCl [pH 7.2]) and V. vulnificus agar (VVA; 2% peptone, 3% NaCl, 1% cellobiose, 0.06% bromothymol blue [pH 8.2]) to enumerate V. parahaemolyticus and V. vulnificus, respectively. Following incubation at 37°C for 16 to 18 h, Whatman 541 ashless filters (Whatman, Kent, ME) were used to lift colonies from each plate. Filters were probed using alkaline phosphatase-conjugated oligonucleotide probes (DNA Technology A/S, Risskov, Denmark) specific for V. parahaemolyticus (*tlh*) or V. vulnificus (*vvhA*); samples collected between 2009 and 2012 were also screened for V. parahaemolyticus virulence markers (*trh* and *tdh*). Probes, listed in Table 3, were visualized using nitroblue tetrazolium chloride/5-bromo-4-chloro-3-indolyl (NBT/BCIP) ready-to-use tables (Roche, Basel, Switzerland), per manufacturer instructions. Probe-positive colonies, i.e., those that were purple-brown, were counted, and the results presented are the averages of duplicate hybridizations. Sterile PBS (1 mL) spread onto T1N3 agar or VVA was used as a blank control. To ensure that the deionized water used to scrub oysters did not contain traces of culturable *Vibrio* bacteria, 1 mL was spread onto T1N3 or VVA during each hybridization. Cultures of V. parahaemolyticus (ATCC 17803 [*tlh*^+^, *tdh, trh*^+^], TX2103 [*tlh*^+^, *tdh*^+^, *trh*], and AQ4037 [*tlh*^+^, *tdh, trh*^+^]), and V. vulnificus (ATCC 27562 [*vvhA*^+^]) were prepared under standard growth conditions in Luria-Bertani broth at 37°C overnight with aeration, and 1 μL was spotted onto T1N3 agar or VVA three times for each hybridization to serve as positive and negative controls, respectively.

**TABLE 3:** Probes used in this study for direct plating and DNA colony hybridization

Description (reference)	Primer or probe	Sequence (5′–3′)^*a*^	Control strain
Thermolabile hemolysin (122)	*tlh*-AP	AAAGCGGATTATGCAGAAGCACTG	V. parahaemolyticus ATCC 17803
Thermostable direct hemolysin (123)	*tdh*-AP	ACTTTGCTTTCAGTTTGCTATTGGCT	V. parahaemolyticus TX2103 (*tdh*^+^ *trh*)^*b*^
Thermostable direct-related hemolysin (124)	*trh*-AP	GGTTCTATTCCAAGTAAAATGTATTTG	V. parahaemolyticus AQ4037 (*tdh trh^+^*)^*b*^
V. vulnificus hemolysin *vvhA* (125)	*vvhA*-AP	GAGCTGTCACGGCAGTTGGAACCA	V. vulnificus ATCC 27562

a All probes were modified by (5′) conjugation with alkaline phosphatase.

b Characterized previously (67).

### Statistical analysis.

Statistical analysis was done using R v.4.2 (118), and figures were generated using ggplot2 (119) and plotly (120). Environmental parameters and DPCH results were compared among locations, seasons, and collection periods. For comparisons of DPCH results, the average number of CFU detected across duplicate hybridizations was used, following normalization to sample volume for water (CFU per milliliter), oyster (CFU/0.1 g), and sediment (CFU/0.1 g). Where appropriate, data were log transformed using the log1p method, i.e., [log(*x* + 1)]. To evaluate the relationships of environmental parameters and DPCH results, surface recordings were used for water samples and bottom recordings were used for oyster and sediment samples. Both individual stations and pooled data were evaluated for both collection periods. However, since samples collected between 2009 and 2012 were representative of >50 sampling events from each of two stations, 15 sampling events were randomly selected from both CR and TS using the “sample” command from the base R software package. To ensure consistency of subsampling across data analysis, the seed was set to seven, which was chosen by random number generation. To test for significance of the observed differences of means between two groups, Wilcoxon test was used, while one-way ANOVA was used to compare differences of means among more than two groups. Linear regression and Kendall’s tau correlation methods were used to quantify the relationship of DPCH results with temperature. A nonparametric regression model, i.e., locally weighted smoothing (locally estimated scatterplot smoothing [LOESS]), was used to visualize the relationship of DPCH results with environmental parameters lacking linear correlation. Box plots were used to summarize distributions by indication of interquartile range. Scatterplots were used to visualize DPCH results relative to environmental parameter values. Normalized kernel density estimates, i.e., a smoothed version of the histogram, were used to visualize the count of samples demonstrating >10 CFU relative to each environmental parameter value.
