# Supplementary material for: Environmental Factors Influencing Occurrence of Vibrio parahaemolyticus and Vibrio vulnificus
Source: Appl Environ Microbiol. 2023 May 24;89(6):e00307-23. doi: 10.1128/aem.00307-23 (PMC10304686; doi:10.1128/aem.00307-23)
Supplement: Supplemental file 1 — Fig. S1. Download aem.00307-23-s0001.pdf, PDF file, 1.5 MB [file aem.00307-23-s0001.pdf]

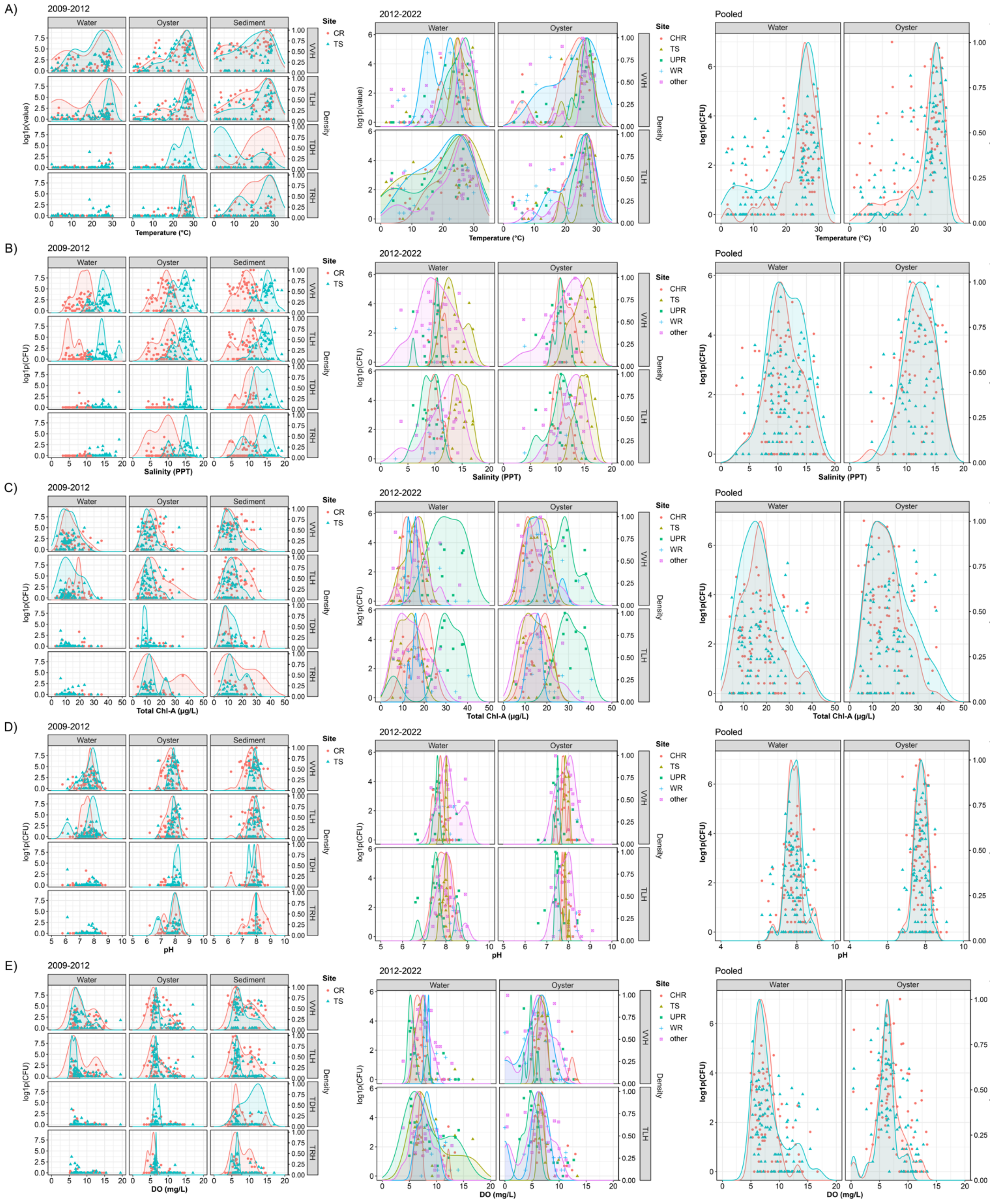

Figure S1: Environmental parameters associated with moderate genetic marker density.

Scatterplots show normalized kernel density of each genetic marker detected above the threshold of 10 CFU per hybridization, with respect to environmental parameter values. “log1p” represents log(x+1). Stations abbreviated as Chester River (CR), Tangier Sound (TS), Choptank River (CHR), Upper Patuxent River (UPR), and Wicomico River (WR). Panels represent samples collected between (left) 2009-2012 and (center) 2019-2022 and (right) pooled data from both collection periods. Environmental parameters are shown as (A) temperature, (B) salinity, (C) total chlorophyll *a*, (D) pH, and (E) DO.
